# Painless thyroiditis associated with SARS-CoV-2 and influenza infections in a patient with central hypothyroidism after pituitary surgery

**DOI:** 10.1530/EDM-24-0037

**Published:** 2024-10-24

**Authors:** Norio Wada, Hajime Sugawara, Ayaka Satoh, Shuhei Baba, Arina Miyoshi, Shinji Obara

**Affiliations:** 1 Department of Diabetes and Endocrinology, Sapporo City General Hospital, Sapporo, Japan; 2 Clinical Training Center, Sapporo City General Hospital, Sapporo, Japan

**Keywords:** painless thyroiditis, severe acute respiratory syndrome coronavirus 2, coronavirus disease 2019, influenza

## Abstract

**Summary:**

We present the case of a 50-year-old Japanese woman who was transferred to our hospital with a 2-day history of fever, sore throat, and malaise. She was diagnosed with acromegaly 9 months ago while being treated for diabetic ketoacidosis, for which she underwent pituitary surgery. She was diagnosed with hypopituitarism postoperatively and was prescribed hydrocortisone and levothyroxine. Her glycemic control was good on metformin. Tests for severe acute respiratory syndrome coronavirus 2 (SARS-CoV-2) and influenza were positive in the emergency room. Other laboratory findings included thyrotoxicosis (free T3: 9.13 pg/mL; free T4: 3.64 ng/dL; and thyroid-stimulating hormone (TSH): <0.01 μIU/mL) and a high C-reactive protein (CRP) level (3.84 mg/dL). The test for the TSH receptor antibody was negative. She had no apparent goiter and reported no tenderness in response to thyroid palpation. 99m-Technetium scintigraphy revealed decreased tracer uptake. Ultrasonography showed no hypoechoic lesions. Her thyrotoxicosis spontaneously resolved after 6 weeks. Although both anti-thyroglobulin antibody (TgAb) and anti-thyroid peroxidase antibody (TPOAb) were negative 9 months ago, TgAb was positive at admission. The test for TPOAb became positive 6 weeks later. These findings were suggestive of painless thyroiditis. In this patient, painless thyroiditis was believed to be caused by SARS-CoV-2 and influenza infections. Screening tests of thyroid function in patients with viral infections such as SARS-CoV-2 or influenza are recommended, even when thyroid gland pain or tenderness is not observed.

**Learning points:**

- We describe a case of painless thyroiditis associated with SARS-CoV-2 and influenza infections.
- Although a few cases of painless thyroiditis associated with COVID-19 have been reported, no cases of painless thyroiditis associated with influenza have been reported.
- In this case, thyrotoxicosis developed immediately after the viral infection. In addition, tests for anti-thyroglobulin antibody and anti-thyroid peroxidase antibody were negative before the onset of symptoms. Tests for the former became positive at the time of onset of symptoms, whereas tests for the latter became positive several weeks after the onset of symptoms.
- Patients with viral infections such as SARS-CoV-2 and influenza, who had no goiter or thyroid tenderness, may develop painless thyroiditis; screening tests for thyroid function are recommended.

## Background

The spread of severe acute respiratory syndrome coronavirus 2 (SARS-CoV-2) began in December 2019 and resulted in the coronavirus disease 2019 (COVID-19). SARS-CoV-2 affects the endocrine system – including the thyroid gland (1), and numerous previous reports have linked subacute thyroiditis (SAT) with COVID-19 (2), Graves’ disease (3), and autoimmune thyroiditis (4). This report describes a patient with diabetes and postsurgical acromegaly who was hospitalized with SARS-CoV-2 and influenza coinfections and demonstrated thyrotoxicosis on admission.

## Case presentation

A 50-year-old Japanese woman was transferred to our hospital with a 2-day history of fever, sore throat, and general malaise. She first visited our hospital because of diabetic ketoacidosis (DKA) 9 months ago. While being treated for DKA, both computed tomography (CT) and magnetic resonance imaging revealed a 16-mm pituitary tumor. Although she demonstrated subtle acromegalic features, her laboratory findings showed high serum growth hormone (GH: 14.4 ng/mL) and insulin-like growth factor-1 (IGF-1: 284 ng/mL). Her serum free T3 (FT3: 0.91 pg/mL), free T4 (FT4: 0.48 ng/dL), and thyroid-stimulating hormone (TSH: 0.04 mIU/mL) levels were low. After improvement of glycemic control through intensive insulin therapy, her serum IGF-1 level increased to 418 ng/mL. She demonstrated no GH suppression during the 75-g oral glucose tolerance test and showed a nadir serum GH level of 18.9 ng/mL (120 min). A thyrotropin-releasing hormone loading test revealed paradoxically increased levels of GH (28.8 ng/mL at 0 min and 35.4 ng/mL at 30 min) and decreased levels of TSH (0.04 µIU/mL at 0 min and 0.22 µIU/mL at 60 min) and prolactin (0.82 ng/mL at 0 min and 1.34 ng/mL at 30 min). A corticotropin-releasing hormone loading test revealed normal levels of adrenocorticotropic hormone (16.2 pg/mL at 0 min and 28.9 pg/mL at 30 min) and cortisol (13.3 mg/dL at 0 min and 20.7 mg/dL at 30 min). A luteinizing hormone (LH)-releasing hormone loading test revealed decreased levels of LH (0.10 µIU/mL at 0 min and 0.38 µIU/mL at 60 min) and follicle-stimulating hormone (1.01 µIU/mL at 0 min and 8.92 µIU/mL at 120 min). She was diagnosed with acromegaly due to a GH-producing pituitary tumor and underwent endoscopic surgery 6 months ago, by which time her GH and IGF-1 levels were normalized. The pathological diagnosis was a GH-producing pituitary neuroendocrine tumor. She developed postsurgical hypopituitarism and was administered hydrocortisone (20 mg/day) and levothyroxine (25 µg/day) for central hypothyroidism demonstrated at her first visit after surgery. Her blood glucose levels were well controlled with metformin (250 mg daily), and her hemoglobin A1c (HbA1c) levels remained at 6.0–7.2%.

When she presented to our hospital on day 0, her body temperature was 39.7°C, her heart rate was 130 beats/min, and her blood pressure was 107/65 mm Hg. She had no goiter and reported no tenderness in response to neck palpation. Screening tests for SARS-CoV-2 (via PCR testing) and influenza (via antigen testing) were positive. Chest CT revealed no signs of pneumonia. She was admitted to an infection unit at our hospital and, later that same day, underwent a screening blood test that incidentally revealed the presence of thyrotoxicosis.

## Investigation

The laboratory results on day 0 revealed thyrotoxicosis (FT3: 9.13 pg/mL; FT4: 3.64 ng/dL; and TSH: <0.01 μIU/mL and a high CRP level (3.84 mg/dL). Her thyroglobulin was normal at 9.21 (reference range (RR): <31 ng/mL). TSH receptor antibody was negative at <0.5 IU/L (RR: <2.0). The test for anti-thyroglobulin (TgAb) was positive at 201.5 (RR: <19.3 IU/mL), and the test for the anti-thyroid peroxidase antibody (TPOAb) was negative at 3.2 (RR: <3.3 IU/mL), although TgAb and TPOAb were negative at <10 and 1.6, respectively, 9 months ago.

**Figure 1:**
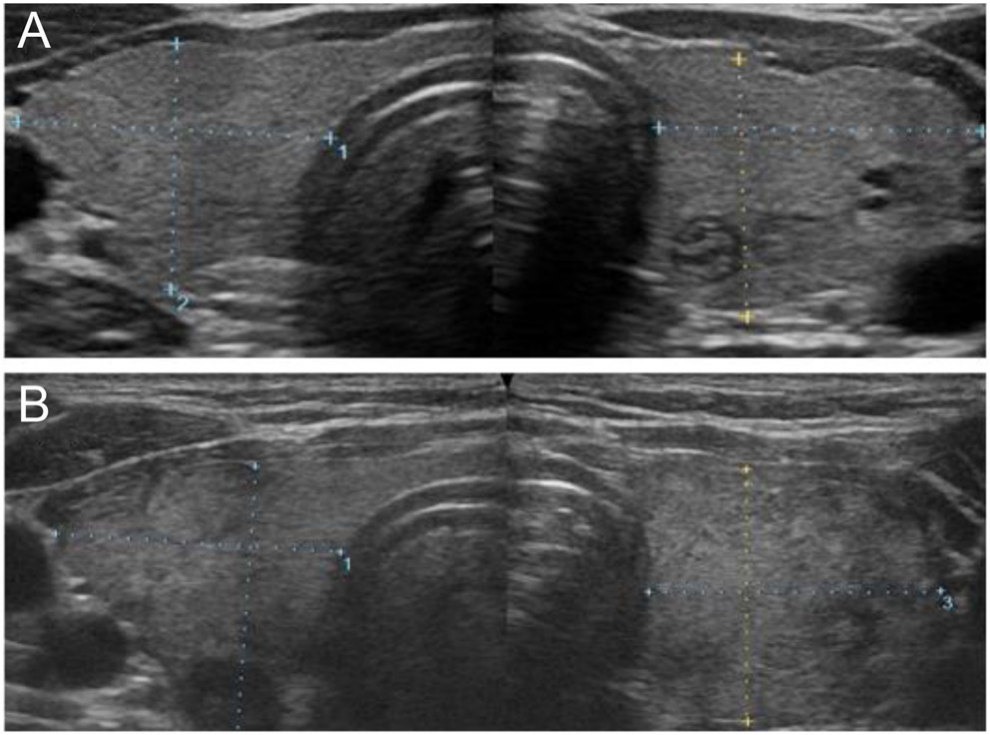
Ultrasonography of the thyroid gland. Ultrasonography of the thyroid gland at diagnosis of acromegaly (A) and admission for SARS-CoV-2 and influenza infection (B).

**Figure 2:**
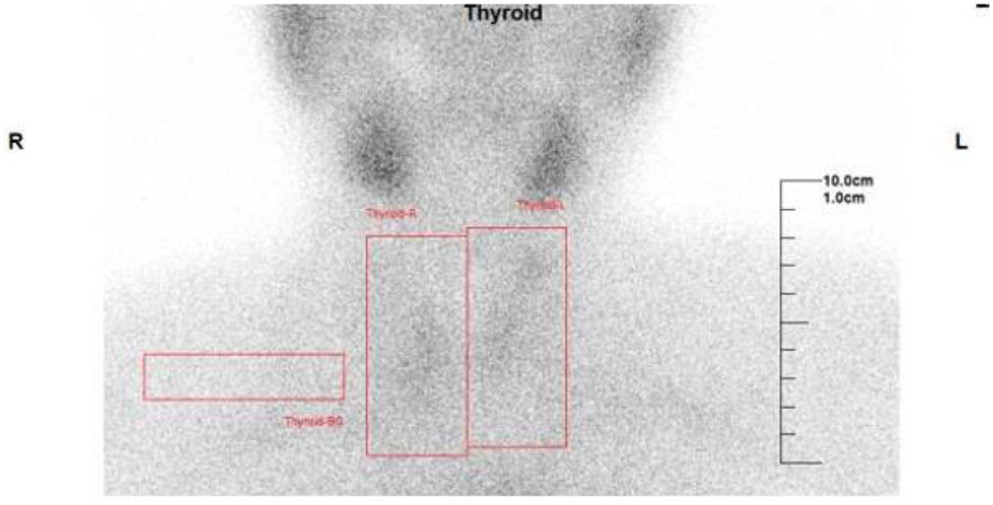
99m-Technetium scintigraphy. 99m-Technetium scintigraphy showed a decreased tracer uptake at 0.4% (reference range: 0.5–4.0%).

Ultrasonography of the thyroid gland was performed twice, once at the diagnosis of acromegaly and then on day 8 at coinfection with SARS-CoV-2 and influenza virus. In the second examination, the thyroid gland’s internal echo pattern was slightly rougher than that observed during the first examination. We also observed no hypoechoic lesions characteristic of SAT (Fig. 1). On day 11, 99m-technetium scintigraphy revealed a 0.4% decrease in tracer uptake in the thyroid gland (RR: 0.5–4.0%) (Fig. 2).

The human leukocyte antigen (HLA) types identified for serum were A2, A3, B3901, B44, Cw5, Cw7, DR13, DR15, DQ6, and DQ6, and for DNA types, they were A*02:06, A*03:01, B*30:01, B*44:02, Cw*05:01, Cw*07:02, DRB1*13:01, DRB1*15:01, DQB1*39:01, and DQB1*44:02.

## Treatment

Peramivir (300 mg) was administered intravenously on the day of admission. Nirmatorelvir (300 mg twice daily) was administered for 5 days. Hydrocortisone was administered intravenously at a dose of 50 mg/day for 3 days, with a later dose reduction to 20 mg/day. While being hospitalized, she received insulin therapy for hyperglycemia. Her fever disappeared on day 6. She was not treated for thyrotoxicosis, except for the administration of propranolol 30 mg/day for 4 days. Two weeks after the onset of the symptoms, she was discharged from our hospital. At 6 weeks after onset, her serum FT3 and FT4 levels normalized, and she resumed levothyroxine supplementation.

## Outcome and follow-up

After the disappearance of thyrotoxicosis, hydrocortisone (20 mg daily) and levothyroxine (25 μg daily were continued. Before pituitary surgery, tests for both TgAb and TPOAb were negative. On day 0, the test for TgAb was positive at 201.5 IU/mL, and TPOAb was negative at 3.2 IU/mL. The test for TPOAb turned positive 6 weeks after onset at 3.6 IU/mL. At 6 and 12 months after SARS-CoV-2 and influenza coinfection, tests for TgAb and TPOAb remained positive with a decreasing trend. Twelve months after the coinfection, her levothyroxine dose was increased to 75 μg/day. Because the glycemic control became worse, dapagliflozin (5 mg/day) was administered, and the metformin dose was increased to 500 mg/day (Table 1).

**Table 1:** The clinical course of the present case.

	Observation period						Reference range
	−9 M	−3 M	At onset	6 Wk	8 M	12 M	
Medication							
Insulin infusion	168 units						
HC, mg		50	20	20	20	20	
LT4, µg			25		50	75	
MET, mg			250		250	500	
DAPA, mg						5	
FT3 (pg/mL)	0.91	1.41	9.13	3.60	1.82	2.51	2.30–4.00
FT4 (ng/dL)	0.48	0.80	3.64	0.90	0.70	1.09	0.90–1.70
TSH (µIU/mL)	0.06	0.03	<0.01	0.01	0.31	0.05	0.62–4.23
TgAb (IU/mL)	<10.0	N.D.	201.5	515.5	117.2	71.7	<19.3
TPOAb (IU/mL)	1.6	N.D.	3.2	3.6	4.1	3.8	<3.3
HbA1c (%)	15.4	7.4	6.6	6.6	7.1	9.4	4.9–6.0

DAPA, dapagliflozin; FT3, free triiodothyronine; FT4, free thyroxine; HbA1c, hemoglobin A1c; HC, hydrocortisone; LT4, levothyroxine; M, months; MET, metformin; N.D., not done; TgAb, anti-thyroglobulin antibody; TPOAb, anti-thyroid peroxidase antibody; TSH, thyroid-stimulating hormone; Wk, weeks.

## Discussion

We reported a case of painless thyroiditis that developed immediately after coinfection with SARS-CoV-2 and influenza virus. In this patient, whose thyroid function had remained normal under levothyroxine after pituitary surgery, mild thyrotoxicosis was detected via screening tests on admission. This finding is thought to be endogenous and unrelated to the levothyroxine that this patient had taken. Except for tachycardia, we observed no symptoms suggestive of hyperthyroidism. Because the test for TRAb was negative and the tracer uptake was decreased on scintigraphy, the patient was considered to have destructive thyroiditis rather than Graves’ disease. Although she had a fever and increased serum CRP levels, there was no pain or tenderness in the thyroid gland, and no hypoechoic area was observed on ultrasonography. HLA typing revealed the absence of HLA-B35, a characteristic commonly observed in patients with SAT (5). Based on this finding, she was diagnosed with painless thyroiditis.

Two cases of painless thyroiditis after SARS-CoV-2 infection have been reported. The first case was of a male in his 50s. He was admitted to the hospital for treatment of COVID-19, and thyrotoxicosis was incidentally diagnosed from a blood test performed at admission (6). Tests for TgAb, TPOAb, and TRAb were negative. He had no symptoms suggestive of thyrotoxicosis and no neck pain. Thyroid scintigraphy showed a marked decrease in tracer uptake in the thyroid gland, and he was diagnosed with painless thyroiditis. On follow-up medical examination, his thyroid function normalized without any specific medication.

The second case was of a woman in her 50s with papillary thyroid cancer and primary hyperparathyroidism (7). The surgery was scheduled for 4 months later, but it was postponed because she was diagnosed with COVID-19. Five months after the first visit, she was admitted to the hospital for a planned thyroid lobectomy and parathyroidectomy. Her blood tests on admission showed thyrotoxicosis, with negative TRAb and thyroid-stimulating antibody. Tests for TgAb and TPOAb, which were initially negative, became positive after SARS-CoV-2 infection. Three days after admission, the patient underwent a left thyroid lobectomy and parathyroidectomy. Histopathological examination confirmed the diagnosis of papillary thyroid carcinoma and parathyroid adenoma. Thyroid tissue showed chronic inflammatory cell infiltration associated with the destruction of thyroid follicles. These findings were consistent with painless thyroiditis.

Similarly, in the present patient, tests for TgAb and TPOAb, which were negative at the diagnosis of acromegaly, turned positive after coinfection with SARS-CoV-2 and influenza virus; however, unlike the second case, the onset of painless thyroiditis was observed immediately after coinfection with SARS-CoV-2 and influenza virus. At least in this patient, the destruction of the thyroid follicles and production of TgAb occurred immediately after the coinfection.

Based on the literature review, we found no report of painless thyroiditis associated with influenza infection. In contrast, two cases of SAT after infection with the influenza virus have been reported (9, 10). Therefore, in the current case, both SARS-CoV-2 and influenza infections are potential causes of destructive thyrotoxicosis.

Regarding the pathogenesis of painless thyroiditis associated with COVID-19, direct damage to thyroid follicular cells by SARS-CoV-2 and indirect damage to thyroid follicular cells via inflammatory cytokines are assumed. Angiotensin-converting enzyme 2, the receptor for SARS-CoV-2, is expressed in thyroid follicular cells (8). Transmembrane serine protease 2, which plays a role in the cell invasion of SARS-CoV-2, is highly expressed in thyroid tissue (11). SARS-CoV-2 infection induces the hyperactivity of immune cells, including Th1/Th17 lymphocytes, through pro-inflammatory cytokines. This phenomenon has also been observed in destructive thyroiditis caused by IFN-α (12). The spike protein and nucleus of SARS-CoV-2 are structurally homogeneous to TPO. Therefore, the antibody against TPO may develop immediately after SARS-CoV-2 infection (13). In contrast, in this patient, TPOAb appeared later than TgAb. The findings of the present patient may provide clues to elucidate the pathogenesis of painless thyroiditis associated with SARS-CoV-2 infection, for which the course of the present case remains unclear.

In the case of Cushing’s syndrome, it is known that the correction of hypercortisolism via adrenalectomy can exacerbate autoimmune thyroid disease (14). In the current case, because a physiological dose of glucocorticoids was administered to compensate for hypopituitarism after pituitary surgery, it is assumed that the immunomodulatory effect of glucocorticoids had no influence on the onset of painless thyroiditis. On the other hand, in the current patient, the absence of an endogenous increase in glucocorticoids immediately after infection may have favored an abnormal immune response against the thyroid gland. Furthermore, we could not find any reports indicating that the correction of GH excess via acromegaly treatment promotes the onset of painless thyroiditis.

In conclusion, painless thyroiditis may develop secondary to infection of SARS-CoV-2 or influenza. Thus, a screening test for thyroid function is useful for patients with viral infections such as SARS-CoV-2 or influenza to detect the presence of thyrotoxicosis, even when they don’t have goiter or thyroid tenderness.

## Declaration of interest

The authors declare that there are no conflicts of interest that could be perceived as prejudicing the impartiality of the study reported.

## Funding

This study did not receive any specific grant from any public, commercial, or not-for-profit funding agency.

## Patient consent

Written informed consent was obtained from the patient for the publication of clinical details and clinical images.

## Author contribution statement

All authors participated in patient treatment, data collection, data interpretation, and manuscript writing and have read and approved the final manuscript.

## References

[1] Mirza SA Sheikh AAE Barbera M Ijaz Z Javaid MA Shekhar R Pal S & Sheikh AB. COVID-19 and the endocrine system: a review of the current information and misinformation. Infectious Disease Reports 2022 14 184–197. (10.3390/idr14020023) 35314653 PMC8938795

[2] Ippolito S Gallo D Rossini A Patera B Lanzo N Fazzino GFM Piantanida E & Tanda ML. SARS-CoV-2 vaccine-associated subacute thyroiditis: insights from a systematic review. Journal of Endocrinological Investigation 2022 45 1189–1200. (10.1007/s40618-022-01747-0) 35094372 PMC8800554

[3] Sousa B Pestana Santos C Gonçalves Ferreira A & Judas T. Graves’ disease caused by SARS-CoV-2 infection. European Journal of Case Reports in Internal Medicine 2022 9 003470. (10.12890/2022_003470) 36051161 PMC9426958

[4] Tee LY Harjanto S & Rosario BH. COVID-19 complicated by Hashimoto’s thyroiditis. Singapore Medical Journal 2021 62 265. (10.11622/smedj.2020106) 32668831 PMC8801861

[5] Stasiak M & Lewiński A. New aspects in the pathogenesis and management of subacute thyroiditis. Reviews in Endocrine and Metabolic Disorders 2021 22 1027–1039. (10.1007/s11154-021-09648-y) 33950404 PMC8096888

[6] San Millán BR Daura MT Ganoza AH & Sala MR. Painless thyroiditis in SARS-CoV-2 infection. Endocrinología, Diabetes y Nutrición 2020 68 757–758. (10.1016/j.endinu.2020.09.001) 33160951 PMC7553067

[7] Nakaizumi N Fukata S Hirokawa M & Akamizu T. Painless thyroiditis incidentally diagnosed following SARS-CoV-2 infection. BMJ Case Reports 2022 15 e252837. (10.1136/bcr-2022-252837) PMC9716833 36455982

[8] Zhou P Yang XL Wang XG Hu B Zhang L Zhang W Si HR Zhu Y Li B Huang CL, *et al.* A pneumonia outbreak associated with a new coronavirus of probable bat origin. Nature 2020 579 270–273. (10.1038/s41586-020-2012-7) 32015507 PMC7095418

[9] Dimos G Pappas G & Akritidis N. Subacute thyroiditis in the course of novel H1N1 influenza infection. Endocrine 2010 37 440–441. (10.1007/s12020-010-9327-3) 20960165

[10] Michas G Alevetsovitis G Andrikou I Tsimiklis S & Vryonis E. De Quervain thyroiditis in the course of H1N1 influenza infection. Hippokratia 2014 18 86–87. 25125962 PMC4103053

[11] Hoffmann M Kleine-Weber H Schroeder S Krüger N Herrler T Erichsen S Schiergens TS Herrler G Wu NH Nitsche A, *et al.* SARS-CoV-2 cell entry depends on ACE2 and TMPRSS2 and is blocked by a clinically proven protease inhibitor. Cell 2020 181 271–280.e8. (10.1016/j.cell.2020.02.052) 32142651 PMC7102627

[12] Ruggeri RM Campennì A Deandreis D Siracusa M Tozzoli R Petranović Ovčariček P & Giovanella L. SARS-COV-2-related immune-inflammatory thyroid disorders: facts and perspectives. Expert Review of Clinical Immunology 2021 17 737–759. (10.1080/1744666X.2021.1932467) 34015983 PMC8182818

[13] Vojdani A Vojdani E & Kharrazian D. Reaction of human monoclonal antibodies to SARS-CoV-2 proteins with tissue antigens: implications for autoimmune diseases. Frontiers in Immunology 2020 11 617089. (10.3389/fimmu.2020.617089) 33584709 PMC7873987

[14] Takasu N Komiya I Nagasawa Y Asawa T & Yamada T. Exacerbation of autoimmune thyroid dysfunction after unilateral adrenalectomy in patients with Cushing's syndrome due to an adrenocortical adenoma. New England Journal of Medicine 1990 322 1708–1712. (10.1056/NEJM199006143222404) 2342537

